# Fibrin glue on an aortic cusp detected by transesophageal echocardiography after valve-sparing aortic valve replacement: a case report

**DOI:** 10.1186/s13256-014-0512-5

**Published:** 2015-03-07

**Authors:** Junko Nakahira, Hisanari Ishii, Toshiyuki Sawai, Toshiaki Minami

**Affiliations:** 1 Department of Anesthesiology, Osaka Medical College, 2-7 Daigaku-machi, Takatsuki, Osaka 569-8686 Japan; 2 Department of Anesthesia, Tenri Hospital, 200 Mishima-cho, Tenri, Nara 632-8555 Japan

**Keywords:** Biological glue, Fibrin glue, Transesophageal echocardiography

## Abstract

**Introduction:**

Fibrin glue is used commonly during cardiac surgery but can behave as an intracardiac abnormal foreign body following surgery. There have been few such cases reported, and they were typically noticed only because of the resulting catastrophic cardiac conditions, such as valvular malfunction. We report a case where, for the first time, transesophageal echocardiography was used to detected fibrin glue that was adherent to the ventricular side of a patient’s aortic valve immediately after aortic declamping.

**Case presentation:**

A 45-year-old Japanese man with Marfan syndrome underwent an aortic valve-sparing operation to treat moderate aortic valve regurgitation resulting from enlargement of his right coronary cusp. Fibrin glue was lightly applied to the suture line between the previous and new grafts. Transesophageal echocardiography performed prior to weaning from the cardiopulmonary bypass revealed mild aortic valve regurgitation in addition to a mobile membranous structure attached to the ventricular side of his aortic valve. It was identified as fibrin glue. We resolved the regurgitation by removing the fibrin glue and repeating the aortic cusp plication. The patient had no complications during recovery.

**Conclusions:**

Fibrin glue can act as an intracardiac foreign body and lead to a potentially fatal embolism. We demonstrated the use of transesophageal echocardiography to detect a fibrin glue-derived intracardiac abnormal foreign body and to confirm its removal. To the best of our knowledge, this is the first case where fibrin glue adherent to the aortic valve was detected by transesophageal echocardiography. These findings demonstrate the importance of using transesophageal echocardiography during cardiac surgery that involves using biological glues.

**Electronic supplementary material:**

The online version of this article (doi:10.1186/s13256-014-0512-5) contains supplementary material, which is available to authorized users.

## Introduction

Various intracardiac abnormal bodies have been reported, including air masses, gauze threads [1,2], and mitral valve chordae entrapped in prosthetic valves [3]. Postoperative valvular malfunction and embolization of the coronary arteries because of biological glue have also been reported and appeared as catastrophic cardiac conditions [4-6]. To the best of our knowledge, ours is the first report of biological glue on an aortic valve fluttering in the cardiac chamber. The biological glue was removed from the aortic valve to avoid it becoming an embolus and transesophageal echocardiography (TEE) was the only approach able to detect the silent abnormality.

## Case presentation

A 45-year-old Japanese man with Marfan syndrome was scheduled for an aortic valve-sparing operation because of moderate aortic valve regurgitation resulting from enlargement of his right coronary cusp. He had previously undergone aortic arch replacement and valvuloplasty of a non-coronary cusp with a patch to correct aortic dissection and moderate aortic valve regurgitation through a tear in the non-coronary cusp.

**Figure 1:**
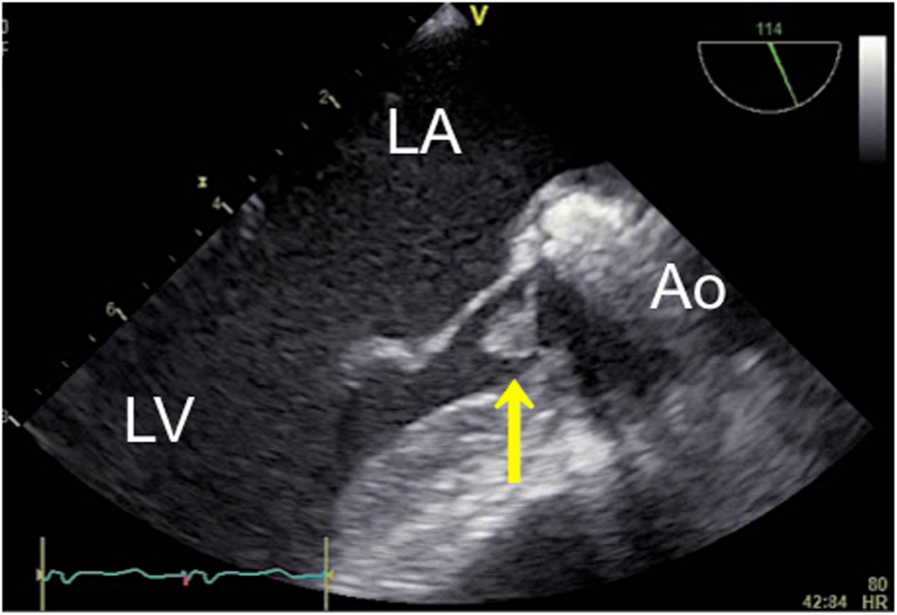
**Two-dimensional transesophageal echocardiography image.** This long-axis view of the aortic valve shows a mobile membranous structure attached to the aortic valve (yellow arrow). Ao, aorta; LA, left atrium; LV, left ventricle.

**Figure 2:**
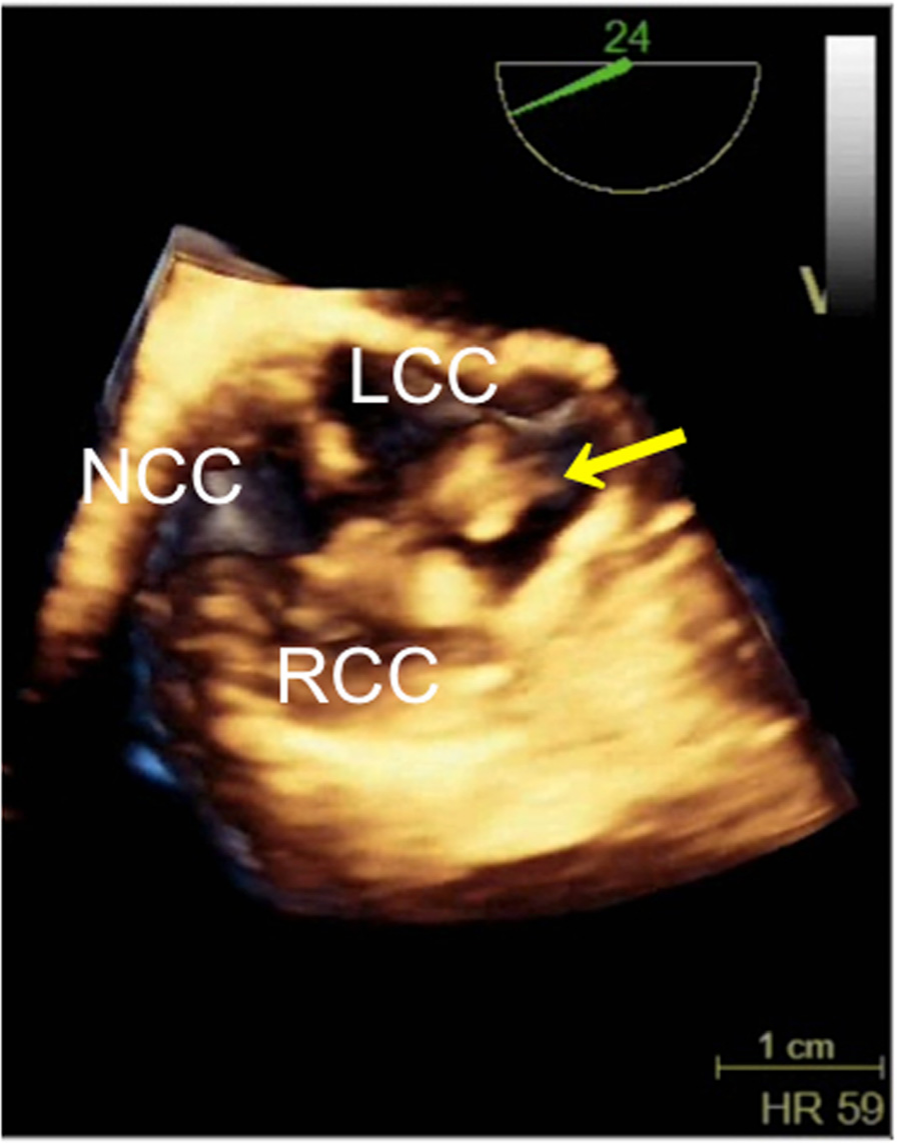
**Three-dimensional transesophageal echocardiography image.** This short-axis view of the aorta shows a mobile structure fluttering in the sinus of Valsalva of the left coronary cusp (yellow arrow). LCC, left coronary cusp; NCC, non-coronary cusp; RCC, right coronary cusp.

**Figure 3:**
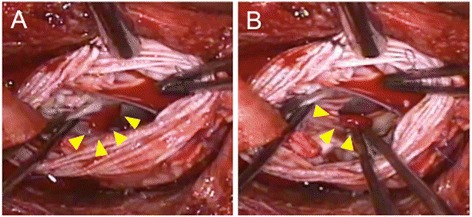
**Photograph of the biological glue.** The arrowheads designate the biological glue that was attached to the ventricular side of the left coronary cusp **(A)** and after easy removal **(B)**.

After induction of anesthesia, an inflow duct for cardiopulmonary bypass (CPB) was placed in his right subclavian artery. Outflow ducts were placed in his superior vena cava and inferior vena cava. An aortic root cannula was inserted into the ascending aortic graft, and the ascending aorta was then clamped. Aortic valve-sparing for root replacement was performed during CPB at 34°C. Mattress sutures with 2–0 Ethibond® (Ethicon, Somerville, NJ, USA) and 4–0 Prolene® (Ethicon) were used for plication of the commissures of the aortic cusps. For central plication of the cusps 6–0 Prolene was used. Mattress sutures with 4–0 Prolene were used for the edge of the artificial aortic graft. The anastomoses between the patient’s sinuses of Valsalva and the graft as well as the coronary arteries and the graft were closed with over-and-over 4–0 Prolene sutures (the latter with felt). Biological glue was applied for hemostasis at the anastomosis sites. It was lightly rubbed and sprayed on the suture line between the previous and new grafts (Vascutek® Gelweave Valsalva™; Terumo, Scotland). Approximately 5mL of Bolheal® (Chemo-Sero-Therapeutic Research Institute, Kumamoto, Japan) was used. Fibrinogen solution (5mL) and thrombin solution (5mL), components of fibrin glue, were also applied. Because of difficulty achieving hemostasis, approximately 5mL of BioGlue® (CryoLife Inc., Kennesaw, GA, USA) was also used. A leak test of the aortic valve with water revealed no leakage on TEE. However, immediately after aortic declamping and before weaning from CPB, TEE detected mild aortic valve regurgitation and a mobile membranous structure attached to the aortic valve on the ventricular side of the valve (Figure 1). A short-axis view of the aortic valve showed that the structure was attached to the left coronary cusp (Figure 2). Two videos show this in more detail (Additional files 1 and 2). We considered the possibility that the abnormal structure was fibrin glue, so the aorta was immediately clamped to avoid arterial embolization of the structure. Following induced cardiac arrest, surgeons opened the anastomoses between the grafts and found a small fibrin clot on the ventricular side of the left coronary cusp (Figure 3). After the fibrin clot was removed, aortic cusp plication was repeated with slight adjustment of the effective heights of the cusps. When weaning from the CPB, we found no further abnormal structures or aortic valve regurgitation. The operating time was 9.5 hours, anesthesia time was 11 hours, and CPB time was 5.5 hours. During the operation we transfused 400mL of autologous blood that had been stored preoperatively, 4 units (560mL) of packed red blood cells, 8 units (960mL) of fresh frozen plasma, 20 units (400mL) of platelets, and 500mL of 5% albumin.

The postoperative course was uneventful. The patient was extubated on the first postoperative day (POD) and was discharged on POD 17. At his 3-month follow-up, echocardiography and computed tomography scans were obtained. He showed neither inflammation nor a pseudoaneurysm at the anastomotic site.

## Discussion

Surgical glue is commonly used during cardiac surgery, especially for surgery of the aorta and aortic valve [7]. In our case, biological glues were not applied for the intracardiac structures but were rubbed and sprayed along the suture lines outside the aortic grafts. We have speculated that the biological glue leaked into the sinuses of Valsalva through the needle holes and attached to the left coronary cusp of the aortic valve.

Postoperative TEE detected the biological glue as an abnormal foreign body. The glue could have embolized a peripheral artery as a left atrial embolus [8-10]. Emboli of limb arteries [11] have been reported previously. Our patient remained stable, and we assumed that weaning from CPB would be uneventful. Being cautious, however, we used TEE to determine if there was an aortic valve leak before weaning. We detected leakage and the biological glue.

Our case illustrates the importance of a comprehensive TEE study during aorta and aortic valve surgery and before weaning from CPB. It is able not only to evaluate the results of the procedure but also to detect foreign bodies and other abnormalities, thereby avoiding postoperative complications.

## Conclusions

Biological glue can become a foreign body in cardiac chambers. Intraoperative TEE is the only approach to detecting this abnormality in a stable situation.

## Consent

Written informed consent was obtained from the patient for publication of this case report and any accompanying images. A copy of the written consent is available for review by the Editor-in-Chief of this journal.

## Additional files

Additional file 1: **Two-dimensional long-axis view of the aortic valve.** A mobile membranous structure was attached to the aortic valve. Ao, aorta; LA, left atrium; LV, left ventricle.

Additional file 2: **Three-dimensional short-axis view of the aortic valve.** A mobile structure flutters in the sinus of Valsalva of the left coronary cusp. Ao, aorta; left coronary cusp; LV, left ventricle; NCC, non-coronary cusp; RCC, right coronary cusp.

## Abbreviations

CPB: Cardiopulmonary bypass
POD: Postoperative day
TEE: Transesophageal echocardiography
